# Longitudinal impact of bladder cancer diagnosis on common psychiatric disorders

**DOI:** 10.1002/cam4.4346

**Published:** 2021-11-12

**Authors:** Ian J. Cooke, Dattatraya Patil, Katherine Bobrek, Vikram Narayan, Viraj Master, Mark Rapaport, Christopher P. Filson, Shreyas S. Joshi

**Affiliations:** ^1^ Department of Urology Emory University School of Medicine Atlanta Georgia USA; ^2^ Winship Cancer Institute Atlanta Georgia USA; ^3^ Department of Psychiatry University of Utah School of Medicine Salt Lake City Utah USA

**Keywords:** bladder cancer, cancer survivorship, mental health

## Abstract

**Background:**

The presence of psychiatric disorders in patients with cancer is associated with increased morbidity and poorer outcomes. We sought to determine the impact of a new bladder cancer diagnosis on the incidence of depression and anxiety.

**Methods:**

We used a database of billing claims (MarketScan®) to identify patients newly diagnosed with bladder cancer between 2009 and 2018. Patients with preexisting psychiatric disorders or use of anxiolytics/antidepressants were excluded. We matched cases to patients without a bladder cancer or psychiatric diagnosis. Our primary outcome was a new diagnosis of depression, anxiety, or use of anxiolytics/antidepressants. Other exposures of interest included gender and treatment received. We used multivariable regression to estimate odds ratios for these exposures.

**Results:**

We identified 65,846 cases with a new diagnosis of bladder cancer (31,367 privately insured; 34,479 Medicare‐eligible). Compared to controls, bladder cancer patients were more likely to develop new‐onset depression/anxiety at 6 months (privately insured: 6.9% vs. 3.4%, *p* < 0.001; Medicare‐eligible: 5.7% vs. 3.4%, *p* < 0.001) and 36 months (privately insured: 19.2% vs. 13.5%, *p* < 0.001; Medicare‐eligible: 19.3% vs. 16.0%, *p* < 0.001). Women (vs. men, privately insured: OR 1.65, 95%CI 1.53–1.78; Medicare‐eligible: OR 1.63, 95%CI 1.50–1.76) and those receiving cystectomy and chemotherapy (vs. no treatment, privately insured: OR 4.94, 95%CI 4.13–5.90; Medicare‐eligible: OR 2.35, 95%CI 1.88–2.94) were more likely to develop significant depression/anxiety.

**Conclusion:**

A new diagnosis of bladder cancer was associated with increased burden of significant depression/anxiety compared with matched controls. Women and patients receiving more radical treatments had higher rates of depression and anxiety.

## Graphical abstract

Using a large, nationally representative sample, we found that a new diagnosis of bladder cancer was associated with increased burden of mental health diagnoses and medication utilization compared with matched controls. Female patients and those receiving more radical treatments for bladder cancer had higher rates of major depressive disorder, generalized anxiety disorder, and psychiatric medication utilization.

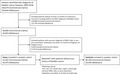

## 1 INTRODUCTION

Mental health disorders, particularly those that are depression and anxiety‐related, are pervasive in the general population of the United States, with an estimated lifetime prevalence of 20.8% for mood disorders and 28.8% for anxiety disorders.^1^ Following a new diagnosis of cancer, there is an increased risk of major depressive disorder (MDD), with rates up to three times that of the general population,^2^, ^3^ as well as increased rates of anxiety and other mental health disorders.^4^ The presence of depression and anxiety in patients with cancer is associated with increased hospital length of stay, decreased acceptance of recommended chemotherapeutic regimens, and increased mortality.^5^, ^6^, ^7^ For patients with bladder cancer, there are increased levels of psychological distress, anxiety, and depressive symptoms prior to radical cystectomy and when receiving adjuvant chemotherapy.^8^, ^9^ Patients with muscle‐invasive bladder cancer and preexisting mental health disorders are significantly less likely to receive guideline‐concordant treatment with worsened disease‐specific and overall survival.^10^ Patients with bladder cancer also have an increased risk of suicide compared with the general population.^11^

Although the impact of psychiatric disorders on bladder cancer outcomes has been described, the downstream impact of a new diagnosis of bladder cancer on the development and severity of mental health disorders is not well understood. This is particularly the case among patients with non‐metastatic and non‐muscle‐invasive disease. Furthermore, prior research has focused on patient self‐reported data to estimate the burden of psychiatric disease in this heterogeneous patient population, rather than the clinical incidence as it related to the delivery of psychiatric care.

To better capture the overall incidence of depression and anxiety disorders in patients with bladder cancer, we queried a large national insurance claims‐based database to identify real‐world diagnoses and psychiatric resource utilization in patients with bladder cancer. The primary objective was to determine the impact of a new diagnosis of bladder cancer on the longitudinal incidence of depression and anxiety. Results from this study will better inform our understanding of the incidence of psychiatric illness in the bladder cancer population.

## 2 METHODS

### 2.1 Dataset

We used enrollment data and administrative billing claims from the Truven MarketScan® Commercial Claims and Encounters and Medicare Supplemental database, a nationally representative sample of over 250 million patients with employer based health insurance in the United States.^12^ This database captures patients with employer sponsored commercial insurance and Medicare Supplemental insurance administered by commercial insurance companies. This database contains enrollment data and administrative billing claims from inpatient and outpatient encounters, as well as pharmaceutical claims. Because the data are deidentified, institutional review board exemption was granted by our institution.

In the United States, the two largest providers of health insurance are programs sponsored by the federal government (Medicare and Medicaid) and private health insurance companies. Any patient above the age of 65, as well as those with disabilities or end‐stage renal disease qualify for Medicare, a federal government‐sponsored insurance program.

### 2.2 Cohort identification

**FIGURE 1:**
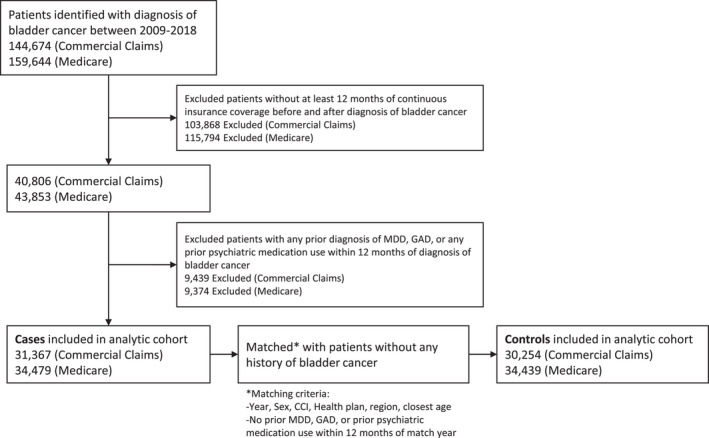
Diagram of analytical cohort generation in commercial claims and medicare population

We identified patients using appropriate diagnosis codes for bladder cancer and procedure codes for treatments for bladder cancer (Table S1). We limited this cohort to patients with 12 months of continuous insurance coverage before and after the date of the claim associated with an initial diagnosis of bladder cancer. We excluded patients with a claim associated with a diagnosis of depression, anxiety, or prescriptions for anxiolytics or antidepressants (Table S2) within the 12 months prior to their bladder cancer diagnosis. We identified a control group comprised of patients without a diagnosis of bladder cancer during the entire study period and also without a previous (12 month) diagnosis of psychiatric disorders. Cases were matched with controls with a 1:1 matching ratio based on gender, Charlson Comorbidity Index (CCI) score, health plan, region, year, and closest age (Figure 1). Control patients were also given an artificial “diagnosis” date corresponding to cases in order to appropriately compare incident rates of psychiatric diagnoses over time.

In the United States, health plans refer to the specific coverage and payment details for patients’ health insurance, and include health maintenance organizations (HMOs), preferred provider organizations (PPOs), exclusive provider organizations (EPOs), point‐of‐service plans (POSs), and high‐deductible health plans (HDHPs). Because different health plans may directly affect patients’ access to and utilization of healthcare, we felt it necessary to match based on health plan given that these access issues may impact a patient's experience with their cancer and subsequent psychiatric comorbidities.

### 2.3 Outcomes of interest

Our primary outcome of interest was a new diagnosis of clinically significant depression and/or anxiety. We defined this based on the presence of claim(s) associated with (a) a diagnosis of MDD or general anxiety disorder or (b) two or more prescriptions typically prescribed for those conditions. We included medication use as part of our combined primary outcome to account for the patients who may have been treated for depressive‐ or anxiety‐related disorders without an ICD code in their record assigning them a specific diagnosis of major depression disorder or generalized anxiety disorder. We required two episodes of medication use to avoid use of single dose benzodiazepines for relatively common minor procedures such as colonoscopy. This decision was based on the assumption that requiring at least two separate episodes of medication administration would limit the amount of peri‐procedural medications that were included.

### 2.4 Exposures of interest

Our primary exposure of interest was a new bladder cancer diagnosis. We compared the incidence of our primary outcome among cases with this exposure and our control group. We also explored the association between the primary outcome of interest and patient gender, as well as its incidence among patients receiving more intense bladder cancer therapy (e.g., radical cystectomy and/or chemotherapy). We subdivided bladder cancer treatments broadly into non‐radical (transurethral resection and intravesical therapies) or radical therapies (chemotherapy, external beam radiation, partial or complete cystectomy) or no treatment.

### 2.5 Statistical analysis

Since we could not ensure individual patients would not be counted twice if they qualified for Medicare over the course of the study period (i.e., a patient with commercial health insurance becoming Medicare‐eligible at age 65) we stratified our analysis based on eligibility for Medicare. We performed bivariate testing to evaluate associations between our outcome of interest and pertinent exposures using generalized chi‐squared testing for categorical and the Wilcoxon rank‐sum test for continuous variables. We fit multivariable logistic regression models to estimate adjusted associations between clinicopathologic variables and our primary outcome of interest among cases. These models were adjusted for year and age at diagnosis, sex, Charlson Comorbidity Index, health plan, region of the country, metropolitan statistical area, and any radical treatment for bladder cancer. Analyses were performed using SAS 9.4 (SAS Institute). Statistical testing was two‐sided with *p* < 0.05 considered statistically significant.

## 3 RESULTS

Our analytic cohorts were comprised of 65,846 patients in total for analysis (*n* = 31,367 with private insurance; *n* = 34,479 with Medicare coverage). Table 1 lists the demographic characteristics of each cohort. Of the overall case cohort, 64,693 were able to be matched to a control (30,254 private insurance; 34,439 Medicare‐eligible). Case and control groups were well matched (Table S3). Median follow‐up time for the privately insured and Medicare‐eligible populations was 29.2 and 32.5 months from diagnosis, respectively.

**TABLE 1:** Demographic characteristics for bladder cancer patients

Covariate	Commercial claims (*N* = 31,367)	Medicare (*N* = 34,479)
Age at diagnosis		
Mean ± Std	53.6 ± 8.9	76.9 ± 7.2
<55	13,353 (42.6)	—
55–59	8460 (27)	—
60–64	9554 (30.5)	—
<75	—	14,067 (40.8)
75–85	—	14,740 (42.8)
>85	—	5672 (16.5)
Gender of patient		
Male	20,220 (64.5)	26,082 (75.6)
Female	11,147 (35.5)	8397 (24.4)
Date year incurred		
2009–13	20,936 (66.7)	24,884 (72.2)
2014–15	5930 (18.9)	6296 (18.3)
2016–18	4501 (14.3)	3299 (9.6)
Charlson Comorbidity Index		
0	6141 (19.6)	1730 (5)
1	3132 (10)	1806 (5.2)
2	9945 (31.7)	6568 (19)
3/+	12,149 (38.7)	24,375 (70.7)
Health plan		
Comprehensive/POS	3950 (13.2)	16,842 (49.7)
HDHP/CDHP	3274 (10.9)	179 (0.5)
HMO	3280 (11)	3360 (9.9)
PPO/EPO	19,417 (64.9)	13,481 (39.8)
Missing	1446	617
Region		
Northeast	8750 (27.9)	9274 (26.9)
North Central	6350 (20.2)	10,364 (30.1)
South	11,337 (36.1)	9271 (26.9)
West	4538 (14.5)	5218 (15.1)
Unknown	392 (1.2)	352 (1)
Metropolitan statistical area		
Yes	26,450 (84.3)	28,183 (81.7)
No	4917 (15.7)	6296 (18.3)

Abbreviations: HDHP/CDHP, high deductible health plan/consumer driven health plan; HMO, health maintenance organization; POS, point‐of‐service; PPO/EPO, preferred provider organization/exclusive provider organization.

**FIGURE 2:**
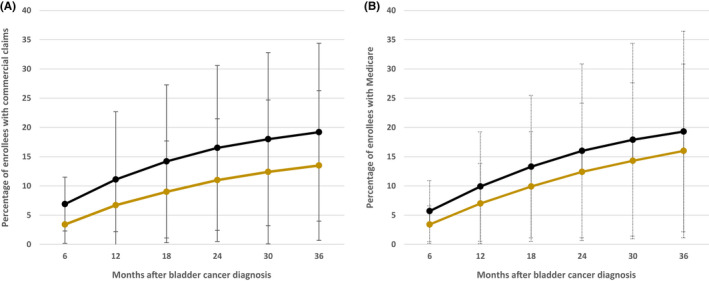
New‐onset diagnoses of MDD or GAD or two or more prescriptions after bladder cancer diagnosis in (A) commercial claims and (B) Medicare population. Black line = bladder cancer cases. Gold line = matched controls

Among the cohort overall, for private insurance and Medicare patients, respectively: 8.7% and 11.1% had claim(s) associated with a diagnosis of depression, 10.6% and 9.4% had claim(s) associated with a diagnosis of anxiety, and 13.8% and 14.5% had two or more prescriptions for medications for depression/anxiety. The incidence of new‐onset clinically significant depression or anxiety among cases and controls over time is shown in Figure 2. We found that bladder cancer patients had substantially higher rates of clinically significant depression/anxiety over 36 months of follow‐up compared with controls (Figure 2A). Among the privately insured bladder cancer patients, the proportion of patients with new‐onset clinically significant depression or anxiety was more than two times that seen among matched controls by 6 months of follow‐up (6.9% vs. 3.4% controls, *p* < 0.001). This finding persisted over 3 years of follow‐up (36 months: 19.2% vs. 13.5% controls, *p* < 0.001). We observed similar patterns among the Medicare‐eligible cohort (6 months: 5.7% vs. 3.4%, *p* < 0.001; 36 months: 19.3% vs. 16.0%, *p* < 0.001) (Figure 2B).

**FIGURE 3:**
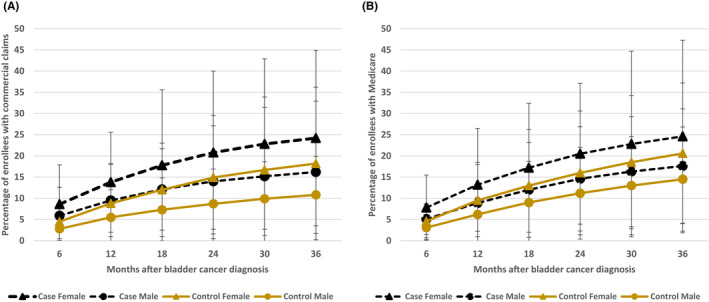
New onset of MDD or GAD or two or more prescriptions after bladder cancer diagnosis in cases and matched controls in (A) commercial claims and (B) Medicare population. Black line with triangle joints = female bladder cancer cases. Black line with circle joints = male bladder cancer cases. Gold line with triangle joints = female matched controls. Gold line with circle joints = male matched controls

Among those with and without a bladder cancer diagnosis, women had significantly higher incidence of clinically significant depression and/or anxiety compared with men. Among the privately insured, 24.2% of women with bladder cancer had newly diagnosed depression or anxiety 3 years after diagnosis compared with 18.2% of women without bladder cancer over a similar time period (*p* < 0.001). At 3 years after diagnosis, 16.2% of men with bladder cancer had new‐onset depression or anxiety, which was similar to the proportion of women without bladder cancer who had new‐onset depression/anxiety over 3 years (18.2%). Similar patterns were seen among the Medicare‐eligible cases and controls at 36 months (Figure 3B).

**FIGURE 4:**
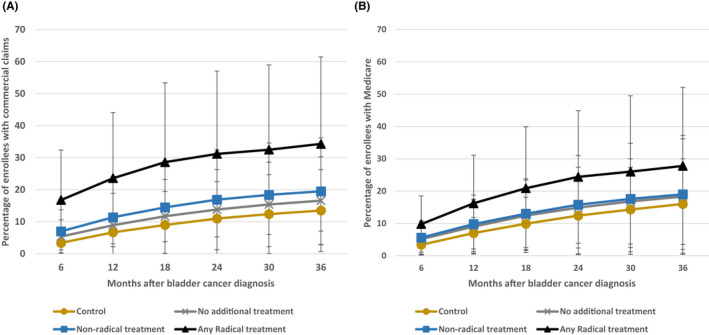
New onset MDD or GAD or two or more prescriptions in cases after bladder cancer diagnosis stratified by treatment modes and in matched controls among (A) commercial claims and (B) Medicare population. Black line with triangle joints = any radical treatment in bladder cancer patients. Blue line with square joints = non‐radical treatment in bladder cancer patients. Gray line with cross joints = no additional treatment in bladder cancer patients. Gold line with circle joints = matched controls

The association between treatments received and the incidence of clinically significant depression/anxiety among cases is demonstrated in Figure 4. We observed a significantly higher incidence of clinically significant depression/anxiety diagnosed among bladder cancer patients who received more radical treatment. At 6 months, privately insured patients receiving radical therapy had a 16.6% rate of clinically significant depression/anxiety which increased to 34.2% at 3 years (*p* < 0.001). At 3 years, bladder cancer patients without radical treatment and matched controls had significantly lower incidence of clinically significant depression/anxiety (19.3% and 13.5%, respectively). A similar pattern was seen among Medicare patients (Figure 4B).

Estimates from our multivariable models are displayed in Table 2. After adjusting for covariates as described in the methods section, privately insured patients aged ≥50 years of age were less likely to develop clinically significant depression or anxiety in the year after bladder cancer diagnosis compared with younger patients (50–61 years: OR 0.83, 95% CI 0.76–0.91; 62–64 years: OR 0.78, 95% CI, 0.70–0.87). Patients with more comorbid diseases were more likely to experience new‐onset depression/anxiety, compared to healthier patients (privately insured: OR 1.54, 95% CI 1.38–1.72; Medicare‐eligible OR 1.31, 95% CI 1.10–1.57). Compared to men, women with bladder cancer were more likely to experience depression/anxiety in the first year after diagnosis (privately insured: OR 1.65, 95% CI 1.53–1.78; Medicare‐eligible OR 1.63, 95%CI: 1.50–1.76), consistent with our findings mentioned previously. Patients receiving chemotherapy and cystectomy (privately insured OR 4.94 vs. no treatment, 95% CI 4.13–5.90; Medicare‐eligible OR 2.35 95% CI: 1.88–2.94) and those receiving only chemotherapy (privately insured OR 3.08 vs. no treatment, 95% CI 2.06–4.60; Medicare‐eligible OR 1.62, 95% CI 1.03–2.55) were more likely to have higher incidence of clinically significant depression or anxiety compared with those without any treatment.

**TABLE 2:** Multivariable analysis results for estimating odds of any new onset of depression, anxiety, or two or more prescriptions after bladder cancer diagnosis within 12 months in commercial claims and Medicare

Covariate	Commercial claims	Medicare
	OR (95% CI)	OR (95% CI)
Age at diagnosis (Ref = <50 for CHI & <72 for Medicare)		
50–61	0.83 (0.76–0.91)	—
61+	0.78 (0.70–0.87)	—
73–80	—	1.02 (0.93–1.11)
80+	—	1.07 (0.98–1.18)
Charlson Comorbidity Index (Ref = 0)		
1	1.15 (1.00–1.34)	1.03 (0.81–1.30)
2	1.22 (1.09–1.36)	1.00 (0.82–1.21)
3/+	1.54 (1.38–1.72)	1.31 (1.10–1.57)
Year of diagnosis (Ref = 2010)		
2011	0.95 (0.83–1.07)	1.08 (0.96–1.21)
2012	1.04 (0.92–1.18)	1.10 (0.98–1.24)
2013	1.03 (0.91–1.18)	1.15 (1.02–1.31)
2014	1.13 (0.98–1.30)	1.29 (1.12–1.48)
2015	1.14 (0.98–1.32)	1.11 (0.95–1.28)
2016	1.20 (1.02–1.40)	1.08 (0.91–1.28)
2017	0.93 (0.78–1.10)	1.03 (0.83–1.29)
Gender (Ref = Male)		
Female	1.65 (1.53–1.78)	1.63 (1.50–1.76)
Health plan (Ref = HMO)		
Comprehensive/POS	1.09 (0.94–1.26)	0.89 (0.78–1.00)
HDHP/CDHP	0.98 (0.84–1.15)	0.57 (0.30–1.09)
PPO/EPO	0.93 (0.82–1.04)	0.88 (0.78–1.00)
Metropolitan statistical area (Ref = No)		
Yes	0.89 (0.80–0.99)	0.95 (0.86–1.05)
Radical treatment for bladder cancer (Ref = No Treatment)		
CTX Only	2.69 (2.26–3.20)	2.00 (1.70–2.35)
CTX+Chemo (GemCis/MVAC)	4.94 (4.13–5.90)	2.35 (1.88–2.94)
Chemo/RT (MitoC/5FU)	3.08 (2.06–4.60)	1.62 (1.03–2.55)
Non‐radical	1.37 (1.27–1.49)	1.11 (1.03–1.20)
Region (Ref = South)		
Northeast	0.84 (0.76–0.92)	0.85 (0.77–0.94)
North Central	0.98 (0.88–1.08)	1.06 (0.97–1.16)
West	1.02 (0.91–1.14)	0.97 (0.86–1.09)
Unknown	1.05 (0.76–1.45)	0.90 (0.62–1.30)

Abbreviations: Chemo/RT, chemotherapy and radiation therapy; CTX, cystectomy; CTX+Chemo, cystectomy and chemotherapy; GemCis/MVAC, gemcitabine and cisplatin or methotrexate, vinblastine, doxorubicin, and cisplatin; HDHP/CDHP, high deductible health plan/consumer driven health plan; HMO, health maintenance organization; MitoC/5FU, mitomycin C or fluorouracil; POS, point‐of‐service; PPO/EPO, preferred provider organization/exclusive provider organization.

## 4 DISCUSSION

### 4.1 Principal findings

In this study of bladder cancer and its impact on the development of depression and anxiety, we report two key findings. First, our study found higher rates of clinically significant depression or anxiety in patients with a new diagnosis of bladder cancer compared with matched controls. Second, among bladder cancer patients, women and those receiving more radical therapies appeared to shoulder the greatest burden of new mental health diagnoses within the first year after a new bladder cancer diagnosis.

### 4.2 Meaning of the study: possible explanations and implications for clinicians

Several studies have linked the diagnosis of bladder cancer with higher rates of mental health disorders and poorer health‐related quality of life.^13^, ^14^, ^15^, ^16^, ^17^ However, there is substantial heterogeneity in study design, cohorts studied, and mental health measurement metrics used within the various studies. As a result, some researchers have found rates of depression and anxiety symptoms of up to 78.0% and 71.3% among bladder cancer patients,^14^ while others only cite rates of 4.7% and 12.5%, respectively.^17^, ^18^ To therefore clarify the “real‐world” magnitude of mental health‐related diagnoses, it is helpful to compare bladder cancer patients to a control group with similar baseline characteristics.

Women are known to be at higher risk than men for anxiety and depressive disorders in general, with prevalence rates of anxiety disorders and depression approximately double that of their male counterparts.^19^, ^20^ Among bladder cancer patients, women with muscle‐invasive bladder cancer receiving adjuvant chemotherapy have been found to have higher self‐reported depression scores compared to men.^8^ On the other hand, men with bladder cancer are more than six times as likely to commit suicide than women.^11^ We observed that, in our cohort, women with a new bladder cancer diagnosis had a higher incidence of clinically significant depression and anxiety compared to men. Interestingly, this gender based difference was large enough that female controls without cancer had similar rates of new‐onset depression and anxiety compared with male patients with bladder cancer. We also found that by 3 years after a diagnosis of bladder cancer, one in four women and one in five men are affected by new‐onset depression or anxiety. Given the substantial prevalence of mental health disorders in this population, providers may able to be provide better care to patients with appropriate attention to these issues.

This study also demonstrated that the type of treatment received may impact the downstream mental health of patients with bladder cancer. Jazzar et al. investigated the burden of psychiatric illness in patients with muscle‐invasive bladder cancer and found that patients who underwent cystectomy with or without chemotherapy were at greater risk for the development of a psychiatric illness than those who received chemotherapy and/or radiotherapy alone; these findings are consistent with our results.^13^ Among those receiving radical therapies in our study, we also found that those receiving cystectomy with neoadjuvant or adjuvant chemotherapy were more likely to develop psychiatric illness compared to those receiving no treatment.

### 4.3 Unanswered questions and future research

Given the substantial amount of morbidity associated with radical treatment for bladder cancer, it is unsurprising that these patients tended to have higher levels of psychiatric illness post‐diagnosis. However, other unmeasured variables may also explain this phenomenon. For example, patients with lower socioeconomic status and patients who are uninsured or have Medicaid have been found to present with more advanced stage tumors compared with privately insured or Medicare patients.^21^ Given the known interactions between socioeconomic status, physical health, and mental health,^22^ other comorbid conditions and social differences may place patients at risk for both development of advanced bladder cancer as well as psychiatric disease. Despite controlling for health plan and Charlson comorbidities in our multivariate analysis, there are likely clinical and sociodemographic moderators that are not adequately captured by these datasets. It is also not clear what role post‐treatment sequelae, such as complications, adverse events from therapy, or cancer recurrence may have contributed to the findings. Future research should seek to explore these areas more closely.

### 4.4 Strengths and weakness of the study

To our knowledge, this MarketScan® cohort of 65,846 cases is the largest matched‐control study of its kind seeking to define the burden of new psychiatric illness in patients with newly diagnosed bladder cancer. By comparison, a recent systematic review examining a similar question found 1659 patients between 13 studies.^17^ One limitation of our study was the inability to extract information about cancer stage and grade from our data. Instead, we used treatment type (non‐radical vs. radical) to subdivide groups into severity of disease. However, this method did not allow for identification of patients with metastatic disease who received non‐curative chemotherapy. On the other hand, the use of this dataset allowed us to cast a wide net and exclude those with both preexisting mental health diagnoses and bladder cancer, allowing for a more “pure” evaluation of patients with new bladder cancer diagnoses who also had no prior depression or anxiety diagnoses within one year. Due to our study design, we may have included some patients with a psychiatric illness greater than 1 year before their diagnosis of bladder cancer, or those with psychiatric illnesses but no claims documentation data in the year prior to cancer diagnosis.

Despite those limitations, we have shown that patients with bladder cancer are at substantially greater risk of developing new psychiatric illness compared to those without bladder cancer, especially among women and those receiving more radical therapies. Increased attention should be given to the mental health of bladder cancer patients post‐diagnosis, as they are at high risk of developing new psychiatric disease, which could meaningfully impact cancer survivorship. Patient questionnaires, multidisciplinary follow‐up clinics, and pathways for referral to appropriate palliative care and/or psychiatric providers and services may facilitate better care for these patients. Additionally, our study raises several important questions and directions for further research, such as the impact of psychiatric comorbidity on survival and on costs of care. Though we have identified some risk factors for the development of clinically significant depression and anxiety, the impact of other important factors, such as the burdens of cancer surveillance or the quality of social support networks, remains unknown.

## 5 CONCLUSION

Patients with newly diagnosed bladder cancer have substantially higher rates of clinically significant depression and anxiety compared to individuals not diagnosed with bladder cancer. This trend emerges soon after diagnosis and is sustained to at least 36 months. Women and those undergoing more radical therapies show the highest burden of incident psychiatric diagnoses. These findings suggest that clinicians should focus any psychiatric intervention early in the disease process and to focus interventions on those who are more likely to undergo radical therapy. Opportunities also exist to address needs specific to female patients. Resources should be allocated to identify these at‐risk patients soon after their diagnoses of bladder cancer.

## CONFLICT OF INTEREST

The authors have no disclaimers or conflict of interest to report.

## ETHICS STATEMENT

Given that all data obtained for use in this study were de‐identified and retrospective, IRB approval was waived.

## Supporting information

Table S1‐S3

## Data Availability

The data that support the findings of this study are available from IBM MarketScan Databases. Restrictions apply to the availability of these data, which were used under license for this study. Data are available at https://www.ibm.com/products/marketscan‐research‐databases/databases or from the authors with the permission of the IBM MarketScan Database.
