# Supplementary material for: Longitudinal impact of bladder cancer diagnosis on common psychiatric disorders
Source: Cancer Med. 2021 Nov 12;10(23):8412–20. doi: 10.1002/cam4.4346 (PMC8633250; doi:10.1002/cam4.4346)
Supplement: Supplementary file 1 — Table S1‐S3 [file CAM4-10-8412-s001.docx]

**Supplementary table 1**: Diagnosis codes and medications used to define the primary outcomes.

| **Health Condition** | **Diagnosis or Procedure Codes** |
| --- | --- |
| **Bladder cancer** | ICD 9: 188x, 233.7 236.7 239.4  ICD 10: C67, D09.0 D41.4 D49.4 |
| **Cystectomy** | Partial – CPT: 51520, 51530, 51550, 51555, 51565  Partial – ICD9CM: 57.6  Radical – CPT: 51570, 51575, 51580, 51585, 51590, 51595, 51596, 51597  Radical – ICD9CM: 57.7, 57.71, 57.79, 68.8 |
| **TURBT** | CPT: 52204, 52214, 52224, 52234, 52235, 52240  ICD9CM: 57.41, 57.49 |
| **Cystoscopy** | CPT: 52000 |
| **Chemo** | J9199, J9201, J9062, J9060, J9000, J9001, J9360, J9250, J9260, J9280, J9190, J9030, J9031, J9357 |
| **Radiotherapy** | CPT: 77373, 77401, 77402, 77407, 77412, 77385, 77386, 77422, 77423, 77427, 77431, 77435, 77499, 77520, 77522, 77523, 77525, 0082T, DW034ZZ, DW035ZZ, DT024ZZ, DT025ZZ, DW064ZZ, DW065ZZ, D7074ZZ, D7075ZZ  ICD: 92.24, 92.25, 92.26 |
|  | |
| **Depression** | ICD 9: 292.2, 296.3X, 298.0, 300.4, 309.1, 311  ICD 10: F32.XX, F33.XX, F34.1, F43.21 |
| **Anxiety** | ICD 9: 300.0X  ICD 10: F41.XX |

**Supplementary table 2:** Medications

| **Health Condition** | **Medications** |
| --- | --- |
| **Depression** | Citalopram, Escitalopram, Paroxetine, Fluoxetine, Fluvoxamine, Sertraline, Desvenlafaxine, Duloxetine, Levomilnacipran, Milnacipran, Tofenacin, Venlafaxine, Vilazodone, Vortioxetine, Etoperidone, Nefazodone, Reboxetine, Viloxazine, Butriptyline, Clomipramine, Desipramine, Dosulepin, Imipramine, Iprindole, Lofepramine, Melitracen, Protripyline, Trimipramine, Opipramol, Tianeptine, Amoxapine, Maprotiline, Mianserin, Mirtazapine, Setiptiline, Isocarboxazid, Phenelzine, Tranylcypromine, Selegiline, Metralindole, Moclobemide, Pirlindole, Toloxatone |
| **Anxiety** | Alprazolam, Buspirone, Chlordiazepoxide, Clonazepam, Clorazepate Dipotassium, Diazepam, Lorazepam, Meprobamate, Midazolam, Oxazepam |

**Supplementary table 3:** Matched selection of controls and bladder cancer cases

| **Covariate** | **Commercial claims** | | **Medicare** | |
| --- | --- | --- | --- | --- |
|  | **Case (N=30,254)** | **Control (N=30,254)** | **Case (N=34,439)** | **Control (N=34,439)** |
| **Age of Patient*** | 53.3 ± 8.9 | 52.9 ± 8.9 | 76.9 ± 7.2 | 76.9 ± 7.2 |
| **Gender of Patient** |  |  |  |  |
| **Male** | 19,148 (63.3) | 19,148 (63.3) | 26,047 (75.6) | 26,047 (75.6) |
| **Female** | 11,106 (36.7) | 11,106 (36.7) | 8,392 (24.4) | 8,392 (24.4) |
| **Date Year Incurred** |  |  |  |  |
| **2009-13** | 19,911 (65.8) | 19,911 (65.8) | 24,873 (72.2) | 24,873 (72.2) |
| **2014-15** | 5,863 (19.4) | 5,863 (19.4) | 6,291 (18.3) | 6,291 (18.3) |
| **2016-18** | 4,480 (14.8) | 4,480 (14.8) | 3,275 (9.5) | 3,275 (9.5) |
| **Charlson Comorbidity Index** | |  |  |  |
| **0** | 6,141 (20.3) | 6,140 (20.3) | 1,729 (5) | 1,729 (5) |
| **1** | 3,131 (10.3) | 3,131 (10.3) | 1,804 (5.2) | 1,804 (5.2) |
| **2** | 9,511 (31.4) | 9,513 (31.4) | 6,563 (19.1) | 6,563 (19.1) |
| **3/+** | 11,471 (37.9) | 11,470 (37.9) | 24,343 (70.7) | 24,343 (70.7) |
| **Health Plan** |  |  |  |  |
| **Comprehensive/POS** | 3,667 (12.7) | 3,667 (12.7) | 16,839 (49.8) | 16,839 (49.8) |
| **HDHP/CDHP** | 3,179 (11) | 3,178 (11) | 162 (0.5) | 162 (0.5) |
| **HMO** | 3,162 (10.9) | 3,162 (10.9) | 3,358 (9.9) | 3,358 (9.9) |
| **PPO/EPO** | 18,914 (65.4) | 18,915 (65.4) | 13,480 (39.8) | 13,480 (39.8) |
| **Region** |  |  |  |  |
| **Northeast** | 8,103 (26.8) | 8,103 (26.8) | 9,269 (26.9) | 9,269 (26.9) |
| **North Central** | 6,200 (20.5) | 6,200 (20.5) | 10,357 (30.1) | 10,357 (30.1) |
| **South** | 11,167 (36.9) | 11,167 (36.9) | 9,261 (26.9) | 9,261 (26.9) |
| **West** | 4,419 (14.6) | 4,419 (14.6) | 5,209 (15.1) | 5,209 (15.1) |
| **Unknown** | 365 (1.2) | 365 (1.2) | 343 (1) | 343 (1) |
| *Mean ± Std | | | | |
